# Supplementary material for: Personalized 3-Gene Panel for Prostate Cancer Target Therapy
Source: Curr Issues Mol Biol. 2022 Jan 15;44(1):360–82. doi: 10.3390/cimb44010027 (PMC8929157; doi:10.3390/cimb44010027)
Supplement: Supplementary file 1 [file cimb-44-00027-s001.zip › cimb-1491912-supplementary.pdf]

current issues in molecular biology

MDPI

Article

Personalized 3-gene panel for prostate cancer target therapy

Sanda Iacobas [1] and Dumitru Andrei Iacobas [2,*]

Supplementary Material

Gene	Description	REV-P	REV-Q	REV-M	REV-Z
LINC0029	long intergenic non-protein coding RNA 294	186.26	167.79	9.72	9.11
RNF180	ring finger protein 180	176.24	88.31	13.00	24.39
ZNF514	zinc finger protein 514	173.86	104.05	18.43	9.59
SCGB1A1	secretoglobin, family 1A, member 1	117.24	192.39	30.93	79.71
ACTA1	actin, alpha 1, skeletal muscle	128.46	188.64	16.90	100.97
TCAP	titin-cap	41.99	185.20	12.58	118.09
OAZ2	ornithine decarboxylase antizyme 2	19.57	12.15	182.91	131.26
ZNF16	zinc finger protein 16	11.25	10.56	164.26	89.80
UBE2I	ubiquitin-conjugating enzyme E2I	22.29	34.84	160.31	195.73
UBE2I	ubiquitin-conjugating enzyme E2I	22.29	34.84	160.31	195.73
MBD6	methyl-CpG binding domain protein 6	16.58	27.71	93.02	162.48
OSCP1	organic solute carrier partner 1	3.82	11.27	54.45	140.84

Gene	Description	REV-A	REV-B	REV-C	REV-N
USP31	ubiquitin specific peptidase 31	188.14	73.66	11.44	16.41
SLC25A47	solute carrier family 25, member 47	184.85	128.52	29.57	79.31
COL11A2	collagen, type XI, alpha 2	183.19	109.21	22.43	20.50
NPY	neuropeptide Y	99.58	131.89	80.91	45.59
HIST2H3A	histone cluster 2, H3a	72.93	146.86	58.95	17.16
MAFK	v-maf avian musculoaponeurotic fibrosarcoma oncogene homolog	10.25	188.55	11.06	7.19
PI16	peptidase inhibitor 16	95.35	38.48	159.77	6.93
SCX	scleraxis basic helix-loop-helix transcription factor	40.88	31.76	189.57	10.23
ZYX	zyxin	24.33	13.72	192.35	30.97
TMCC2	transmembrane and coiled-coil domain family 2	105.67	72.80	12.67	132.20
TTC13	tetratricopeptide repeat domain 13	18.03	40.03	18.09	132.80
UXT	ubiquitously-expressed, prefoldin-like chaperone	117.50	73.66	8.84	133.35

Table S1: The 3 genes most variably expressed (high REV) across biological replicas in all profiled regions of the two patients. Note that, excepting *UBE2I,* common for nodule "M" and normal tissue "Z", there is no overlap of the most variably expressed 3 genes in each region.
